# Advanced Analysis of Diffusion Tensor Imaging Along With Machine Learning Provides New Sensitive Measures of Tissue Pathology and Intra-Lesion Activity in Multiple Sclerosis

**DOI:** 10.3389/fnins.2021.634063

**Published:** 2021-05-07

**Authors:** Olayinka Oladosu, Wei-Qiao Liu, Bruce G. Pike, Marcus Koch, Luanne M. Metz, Yunyan Zhang

**Affiliations:** ^1^Department of Neuroscience, Faculty of Graduate Studies, University of Calgary, Calgary, AB, Canada; ^2^Hotchkiss Brain Institute, University of Calgary, Calgary, AB, Canada; ^3^Department of Clinical Neurosciences, Cumming School of Medicine, University of Calgary, Calgary, AB, Canada; ^4^Department of Radiology, Cumming School of Medicine, University of Calgary, Calgary, AB, Canada

**Keywords:** single-shell high angular resolution diffusion imaging, diffusion tensor imaging, tractography, support vector machine, lesions, intra-lesion pathology

## Abstract

Tissue pathology in multiple sclerosis (MS) is highly complex, requiring multi-dimensional analysis. In this study, our goal was to test the feasibility of obtaining high angular resolution diffusion imaging (HARDI) metrics through single-shell modeling of diffusion tensor imaging (DTI) data, and investigate how advanced measures from single-shell HARDI and DTI tractography perform relative to classical DTI metrics in assessing MS pathology. We examined 52 relapsing-remitting MS patients who had 3T anatomical brain MRI and DTI. Single-shell HARDI modeling yielded 5 sub-voxel-based metrics, totalling 11 diffusion measures including 4 DTI and 2 tractography metrics. Based on machine learning of 3-dimensional regions of interest, we evaluated the importance of the measures through several tissue classification tasks. These included two within-subject comparisons: lesion versus normal appearing white matter (NAWM); and lesion core versus shell. Further, by stratifying patients as having high (above 75%^*ile*^) and low (below 25%^*ile*^) number of MS lesions, we also performed 2 classifications between subjects for lesions and NAWM respectively. Results showed that in lesion-NAWM analysis, HARDI orientation distribution function (ODF) energy, DTI fractional anisotropy (FA), and HARDI orientation dispersion index were the top three metrics, which together achieved 65.2% accuracy and 0.71 area under the receiver operating characteristic curve (AUROC). In core-shell analysis, DTI mean diffusivity (MD), radial diffusivity, and FA were the top three metrics, and MD dominated the classification, which achieved 59.3% accuracy and 0.59 AUROC alone. Between patients, FA was the leading feature in lesion comparisons, while ODF energy was the best in NAWM separation. Collectively, single-shell modeling of common diffusion data can provide robust orientation measures of lesion and NAWM pathology, and DTI metrics are most sensitive to intra-lesion abnormality. Combined analysis of both advanced and classical diffusion measures may be critical for improved understanding of MS pathology.

## Introduction

Multiple sclerosis (MS) is a severe central nervous system disease impacting > 2.8 million people worldwide (Msif Tmsif., 2020). Focal lesions are the hallmark of MS pathology, characterized by several changes, including demyelination, axonal injury, and inflammation (Reich et al., 2018). In addition, MS pathology alters tissue integrity not only in the normal-appearing tissue, but also within the territory of established lesions, leading to heterogeneous damage as seen in chronic active lesions. Ongoing tissue damage in the latter is believed to play a major role in the relentless progression of patient disability in MS (Reich et al., 2018). However, robust measurements of the complex types of MS pathology are still missing. Diffusion magnetic resonance imaging (dMRI) serves as a promising tool for *in vivo* assessment of tissue microstructure (Inglese and Bester, 2010). Moreover, with advances in dMRI techniques, multi-dimensional analysis of tissue properties becomes possible, including localized analysis of lesion activities concerning specific white matter tracts (Inglese and Bester, 2010).

Diffusion tensor imaging (DTI) is a common dMRI method that reflects the orientational dependence of diffusion associated with individual image voxels (Tournier, 2019). Diffusion tensor imaging uses a tensor model, which defines diffusion anisotropy according to three perpendicular diffusion axes originating from a voxel (Tournier, 2019). Several studies have shown that the mean diffusivity (MD) and fractional anisotropy (FA) of DTI detect demyelination and axonal loss in MS, which differentiates lesions from the normal-appearing white matter (NAWM) (Schmierer et al., 2007). Within the lesion context, there is evidence showing increased MD in the lesion core versus perilesional white matter (Klistorner et al., 2018). However, there are no systemic studies of core versus periphery of lesions in MS, and use of a single tensor model in DTI, which does not provide compartmental information, limits its ability to detect specific processes of neuronal pathology. High angular resolution diffusion imaging (HARDI) tackles this problem by using advanced models which enable intra-voxel analysis through increased angular sampling of diffusion signals acquired typically with different diffusion weightings (multi-shell HARDI) (Descoteaux, 2015). A range of multi-shell HARDI models exist, divided mainly by the approach used to model the properties of nerve fibers, such as the cylinder model used in ActiveAx (Alexander et al., 2010), and the stick model in neurite orientation dispersion and density index (NODDI) (Zhang et al., 2012). But acquiring multi-shell HARDI data is not always practical due to time constraints, particularly in a clinical setting.

An alternative approach to multi-shell HARDI is modeling densely sampled data acquired using one diffusion weighting, namely, single-shell HARDI (ssHARDI) (Taquet et al., 2013). In general, dMRI acquired with gradient sampling orientations ≥ 45 is sufficient for this approach (Descoteaux, 2015). Current investigations of ssHARDI mainly focus on its neurite orientation characterization potential (Taquet et al., 2013). While some work has explored the possibility of generating additional compartmental metrics (Grussu et al., 2014; Magnollay et al., 2014), the ability of ssHARDI for advanced intra-voxel analysis of tissue properties remains unclear, particularly based on typical DTI data.

In addition to advances in diffusion modelling, there have also been considerable improvements in tractography investigations based on dMRI (Tournier, 2019). Traditionally, individual white matter tracts (e.g., corticospinal tract) traced by streamlines of tractography form the mainstay for localized analysis of diffusion metrics. However, the local orientation information represented directly by the streamlines may be also critical indicators of tissue properties, including streamline counts and streamline termination frequency, providing a new dimension of microstructural measurements (Ukmar et al., 2012).

The purpose of this study was to test the feasibility of ssHARDI using clinically available dMRI, and investigate how advanced metrics from ssHARDI and DTI tractography compare to traditional DTI measures in assessing MS pathology. The investigations used a machine learning technique, support vector machine (SVM), to evaluate feature importance through several tissue classification tasks. Specifically, based on 3D regions of interest (ROIs), there were two classifications within individual patients: (1) lesion versus the corresponding contralateral NAWM, to obtain a basic understanding of the sensitivity of the features to MS pathology; and (2) lesion core versus shell, to evaluate the detectability of intra-lesion pathology by the features. Between patients, there were also two analyses: lesion comparisons, between patients having high (top 25%) and low (bottom 25%) lesion counts as a surrogate for disease activity; and NAWM comparisons, between the same subject groups.

## Materials and Methods

### Sample

This study evaluated brain magnetic resonance imaging (MRI) scans from a convenience sample of 52 participants with relapsing-remitting multiple sclerosis (RRMS) who were screened for participation in a clinical trial of domperidone as a potential myelin repair agent (ClinicalTrials.gov Identifier: NCT02493049). Participants required at least one gadolinium-enhancing lesion on a screening brain MRI to be eligible for randomization to treatment in the clinical trial. In the present study, we utilized the screening brain MRI scans of participants who were not eligible to continue in the trial as they did not contain any enhancing lesions. In addition, all MRI pulse sequences used in this study were conducted before gadolinium use, and therefore no contrast interference. Written informed consent was obtained from all participants following study approval by the Conjoint Health Research Ethics Board of the University of Calgary.

### Imaging Protocol

All participants had brain anatomical and diffusion MRI undertaken at a 3T scanner (GE Healthcare, Discovery MR750, Milwaukee, United States). Anatomical MRI included T2-weighted and FLAIR images with repetition time (TR) = 6000/7000, echo time (TE) = 100/126 ms, matrix = 512 × 512, and slice thickness = 3 mm without gap; and T1-weighted images with TR/TE = 8.2/3.2 ms, matrix = 256 × 256, field of view (FOV) = 25 × 25 cm, and slice thickness = 1 mm. The dMRI acquisition used an echo planar sequence where b = 1000, 3 b0 volumes, and 45 directions; TR/TE = 8000/61 ms; matrix = 120 × 120, with 2 mm (Inglese and Bester, 2010) isotropic voxels; and FOV = 24 × 24 cm.

### 3D ROI Development

**FIGURE 1:**
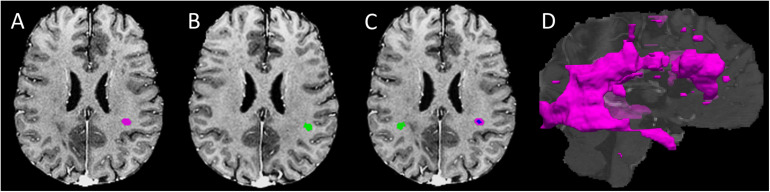
Regions of interest development. **(A)** An example lesion region of interest (ROI) in the T1- FLAIR MRI space (purple mask); **(B)** A mirror-image of the T1-weighted MRI for developing the contralateral normal appearing white matter (NAWM) ROI (green mask) of the lesion shown in **panel A**; **(C)** the finalized contralateral NAWM ROI of the lesion produced using (**panel A, B**), along with converse co-registration between the corresponding image volumes; the blue and purple areas within the lesion represents the core and shell ROIs, respectively; and **(D)** 3D whole brain lesion masks produced by grouping adjacent ROI voxels within and between slices.

To improve the analysis of contextual information, we derived 3D ROIs for all tissue regions, done initially using anatomical MRI (Figure 1).

#### Lesion ROIs

Using the FSL library (Oxford, United Kingdom), all MRI volumes were skull-extracted, and the T2 and FLAIR images were then rigid-body co-registered to T1 images to optimize quality and alignment (Jenkinson and Smith, 2001; Jenkinson et al., 2002; Jenkinson et al., 2012). Lesion segmentation focused on brain white matter, using an automatic toolbox (LST, v3.0.0, SPM12) (Schmidt et al., 2012). Initially, the LST generated a whole-brain lesion map per subject based on co-registered FLAIR and T1 volumes. The lesion map then underwent manual correction using ImageJ (NIH, v1.52j) by referencing the co-registered FLAIR and T2 images. Any area that contained a confluent lesion, showing signal inhomogeneity but with pixels staying connected, was considered a single ROI. Subsequently, lesion ROIs were colocalized across slices using the ‘cluster’ command in FSL with 26-connectivity to obtain 3D ROIs, which were further sorted by ROI size.

#### Contralateral NAWM ROIs

The NAWM ROIs were essentially the mirror image of the lesion ROIs established above. To ensure validity, we applied several quality-control steps. The first step was lesion-filling (Battaglini et al., 2012) in the referencing T1 MRI, followed by creation of a left-right flipped mirror image of the volume. The accuracy of volume flipping was ensured through a cross-correlation-based nonlinear co-registration process between the reference and mirror T1 volumes, using the “SyN” option in the ANTs software (Avants et al., 2008), from which a left-right transformation was obtained. Applying the transformation to the generated lesion masks made the latter geometrically matched to the mirror volume of the reference T1. After left-right flipping, the transformed lesion masks became the “raw” contralateral NAWM ROIs. The next step was refinement of the NAWM ROIs, including eliminating areas overlapping with any lesion region. This step ensured that each 3D NAWM ROI corresponded to each unique 3D lesion ROI with no contamination by tissues of the other type (see Figure 1).

#### Lesion Core and Shell ROIs

The core-shell segmentation focused on lesion ROIs that were large enough to encapsulate a 3 × 3 × 3 cube of voxels. Specifically, defining a lesion core ROI used a 3D volumetric erosion process applied to an eligible lesion mask, which allowed to retain only the central voxels not in contact with any non-lesion background area. Then, subtracting the core from the full lesion ROI produced the single-voxel-thick shell ROI for each lesion.

### dMRI Analysis

#### Pre-processing and DTI Analysis

The dMRI data first underwent eddy current correction (Andersson and Sotiropoulos, 2016). To limit variations, we averaged the 3 b0 volumes and then co-registered the mean volume to the T1 structural space. The resulting mean b0 volume acted as a reference in transforming all calculated diffusion maps to the same T1 space prior to quantitative evaluations. DTI calculation used the FDT procedure in FSL, which provided 4 classical outcomes: MD, FA, axial diffusivity (AD), and radial diffusivity (RD).

#### ssHARDI Analysis

**FIGURE 2:**
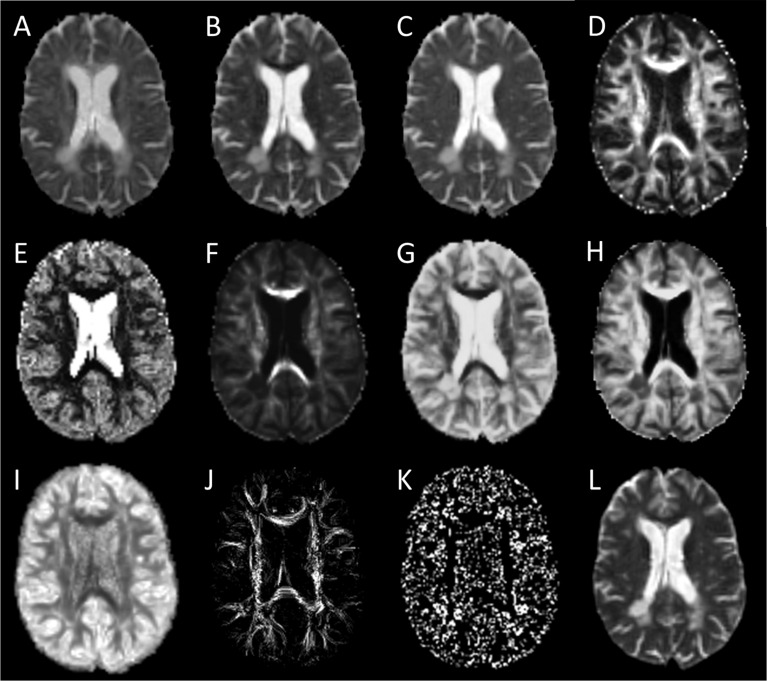
Sample diffusion metrics based on DTI, ssHARDI, and DTI tractography models. Shown are measures from DTI: **(A)** axial diffusivity, **(B)** radial diffusivity, **(C)** mean diffusivity, and **(D)** fractional anisotropy; ssHARDI: **(E)** orientation dispersion index, **(F)** density, **(G)** diameter, **(H)** intracellular volume fraction, and **(I)** orientation distribution function (ODF) energy; and DTI tractography: **(J)** fiber density index and **(K)** fiber termination index. Shown in **(L)** is an example b0 image from DTI for reference.

Given the relatively high angular nature of our dMRI acquisitions, we also explored ssHARDI modeling. In particular, to improve computing efficiency, we used a new modeling method: Accelerated Microstructure Imaging via Convex Optimization (AMICO), which generated equivalent measures to ActiveAx and NODDI (Daducci et al., 2015). Our AMICO outcomes included axonal diameter, axonal density, and intracellular volume fraction (ICVF) from ActiveAx, and orientation dispersion index (ODI) from NODDI (Alexander et al., 2010; Zhang et al., 2012). In addition, to further probe intravoxel orientation information beyond ODI, we calculated the orientation distribution function (ODF) of diffusion (Descoteaux et al., 2007). This in turn enabled us to generate a new parameter termed ODF energy (Figure 2), which referred to the energy of diffusion oriented at individual directions. The energy was calculated as log (p^2), where p represented a collection of probabilities of diffusion magnitude observed at all possible directions. The probabilities were obtained by fitting the diffusion magnitude values from each direction to a normal distribution.

In addition, to test the feasibility of using AMICO to evaluate ssHARDI, we performed an additional experiment to compare outcomes from different datasets. This included: (1) single-shell, 2-shell, and 3-shell HARDI data freely available online from the Human Connectome Project (HCP) (Van Essen et al., 2012); and (2) single-shell data from our own study. The comparisons were done both visually and quantitatively with a concentration on 3 ssHARDI measures out of AMICO ActiveAX: axonal diameter, axonal density, and ICVF. NODDI ODI had been shown to be similar between calculations of single- and multi-shell data (Zhang et al., 2012). In quantitative assessment, we computed the variance of the aforementioned measures at a regional level in 10 example white matter structures bihemispherically: forceps minor, forceps major, genu and splenium of the corpus callosum, and posterior limb of the internal capsule. Each ROI was sized 4 × 4 pixels, which was the maximal uniform dimension that could be fitted within these structures. Statistical analyses were conducted using one-way ANOVA followed by correction for multiple comparisons where applicable.

#### DTI Tractography Analysis

We performed tractography based on DTI using the Diffusion Toolkit (MGH GCRC, United States) (Wang and Wedeen, 2007), which applied a deterministic algorithm, fiber assignment by continuous tracking (FACT) (Wang and Wedeen, 2007). Streamline propagation followed a 35° angular threshold as recommended.

Using the rigid-body transformation matrix derived above from FSL, we also aligned the tractography to the T1 structural space. Based on the TrackVis method (v0.6.1) (Wang and Wedeen, 2007), we evaluated two main tractography measures (see Figure 2): streamline density index (FDi) and streamline endpoint index (FTi), indicating the counts of streamlines passing through or terminating in each voxel, respectively. Our in-house experiment showed that tractography features based on DTI were similar to ssHARDI, so we focused on DTI only here.

### SVM Analysis

In feature ranking, we split the whole dataset randomly into 10 folds (portions), using 9 folds at a time, and repeated 10 times. In classifications, 10-fold cross-validation was performed, with 9 folds for training, and the 10th held-out fold for testing, in each iteration. As a standard practice, we also normalized all feature outcomes to the range 0–1.

We applied a linear SVM to rank the diffusion features through recursive feature elimination (SVM -RFE) (Guyon et al., 2002), with the regularization term fixed to one for optimal model generalization. The squared weights of each attribute of the SVM served as the ranking criteria such that a feature with the smallest weight ranked the lowest. The average ranking of a feature from all model constructions (100 in total) represented the final ranking of the feature.

To assess the contribution of each feature to model classifications, we developed another linear SVM with iterative construction (SVM-IC), achieved by adding features to the model one-by-one from the highest to the lowest rankings. With each feature added, the model is reconstructed through 10-fold cross validation. This provided data to build the rank aggregated receiver operating characteristic (ROC) curves for each combination of successively ranked features, and compute evaluation metrics including the area under the ROC (AUROC) and accuracy, averaged from all tests.

### Patient Stratification

To explore how the imaging features could differentiate patient groups, we stratified the participants as having high and low number of MS lesions as a surrogate of disease activity. The lesion numbers from individual patients were ranked by percentiles. To maximize the likelihood of detecting potential differences, our analysis focused on two patient groups that had highly different lesion numbers: one with the most lesions (≥35) that ranked above the 75%^*ile*^, and the other with the least lesions (≤15) that ranked below the 25%^*ile*^. Group comparisons were done for both lesion tissue (whole lesion ROIs) and NAWM regions, using similar SVM strategies as described above for feature ranking and tissue classification.

### Statistical Analysis

Assessment of all outcomes used the Scikit-Learn package in Python (v0.22.1) and R (v3.6.3). Comparing model performance used the McNemar’s test (Agresti, 1990) for accuracy, and DeLong’s test (DeLong et al., 1988) for AUROC, including model against chance, and between models successively generated.

## Results

### Sample Characteristics

**FIGURE 3:**
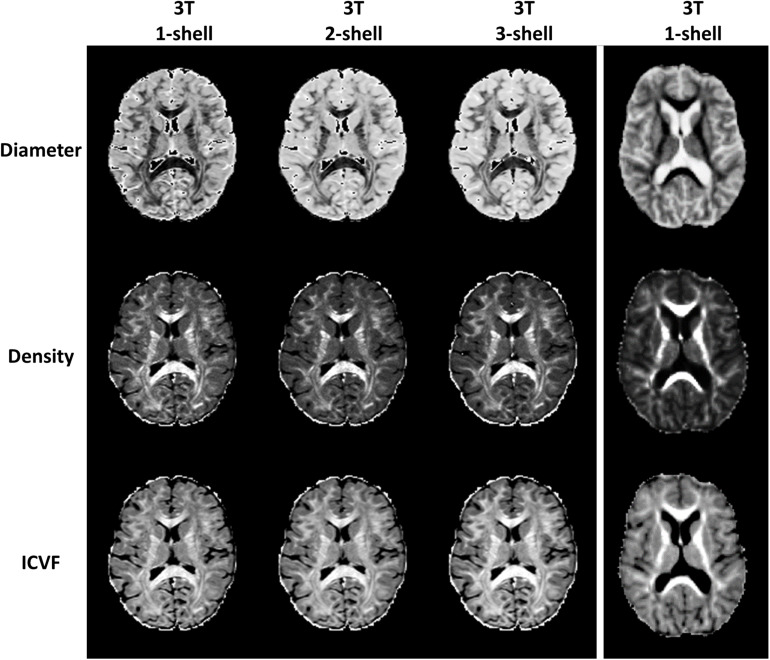
Outcome comparison between single-shell and multi-shell acquisitions. Shown are sample HARDI outcomes based on the ActiveAx model implemented in AMICO using the online Human Connectome Project data (left columns): *b* = 1000, *b* = 1000 and 2000, and *b* = 1000, 2000, and 3000 for the 1-, 2-, and 3-shell, respectively; and using our own diffusion data in this study based on ssHARDI (right column), with *b* = 1000. All represent *in vivo* datasets.

Of the 52 participants, the age range was 18–60 years, Expanded Disability Status Scale was 0–5.5; 36 were women. In total, we identified 2139 lesion ROIs, 4 to 169 per subject; 1560/2139 lesions had matching contralateral NAWM ROIs, 1 to 119 per subject. Among the 1560 lesions, 243 had core-shell analysis (Table 1). In addition, 13 participants had ≥ 35 lesions, totaling 818 lesions, and 12 participants had ≤ 15 lesions, totaling 119 lesions. Similarly, there were 818 and 119 matching contralateral NAWM ROIs in each patient group respectively. Further, example outcomes from ssHARDI modeling appeared similar to that from multi-shell data, including the measures from our own DTI scans (Figure 3). Quantitatively, there were no significant differences in variance for diameter, density, or ICVF (*p* = 0.75, 0.18, and 0.11, respectively) between the different shell calculations using HCP data or data from our own study (Table 2). Further exploration using paired Student’s *t*-tests showed that there were also no significant differences (*p* > 0.05) between the 1-shell measures from HCP and our DTI data in variance of any of the assessed variables following ssHARDI modeling. In total, there were 11 features calculated from all diffusion models.

**TABLE 1:** Lesion counts and volumes by group.

**Lesion ROIs**	**Number**	**Lesion volume (mm^3^)**			
		**Mean**	**Standard error**	**Min**	**Max**
All	2139	256.21	25.65	3.81	16443.64
NAWM-paired	1560	45.41	1.86	3.81	929.88
Core-shell	243	158.14	8.34	35.29	929.88
Shell	–	144.89	6.93	34.33	735.35
Core	–	13.25	1.79	0.95	243.20

*ROI, regions of interest; NAWM, normal appearing white matter.*

**TABLE 2:** Comparison of variance between measures from different shells and datasets computations.

	**HCP1**	**HCP2**	**HCP3**	**In-house**	**ANOVA *p*-value**	**ssHARDI *p*-value**
Diameter	1.416713	1.313007	1.449823	0.939283	0.749132	0.131976
Density	3.62E-06	2.94E-06	3.69E-06	8.1E-06	0.175857	0.192801
ICVF	0.006766	0.006788	0.006341	0.001743	0.114337	0.050831

*HCP1-3, Human connectome project data from 1 to 3 shell acquisitions; In-house, our own “1-shell” DTI data; ssHARDI p-value, Student’s *t*-tests for variances between HCP1 and In-house data.*

### Lesion-NAWM Analysis

**FIGURE 4:**
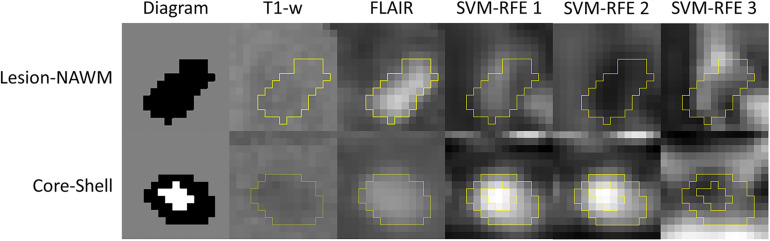
Example lesion regions and top diffusion metrics identified in the tissue separation processes. Shown are diagrams for a whole lesion (black, top) and a core-shell lesion (white and black, bottom) used for the lesion versus NAWM and core versus shell classifications, as well as corresponding appearances in the T1-weighted and FLAIR MRI (columns 1–3). The other columns (4–6) demonstrate the top three diffusion features selected by the recursive feature elimination algorithm (SVM-RFE) in respective classification tasks, which are orientation distribution function energy, fractional anisotropy, and orientation dispersion index (top); and mean diffusivity, radial diffusivity, and fractional anisotropy (bottom).

Tissue alignment metrics ranked higher than magnitude metrics. Specifically, the top three rankings were: HARDI ODF energy, DTI FA, and HARDI ODI (Figure 4). Tractography FDi (fourth), DTI AD (fifth), and tractography FTi (sixth) all ranked within the top half of the 11 features, relatively higher than the other DTI and ssHARDI features.

**FIGURE 5:**
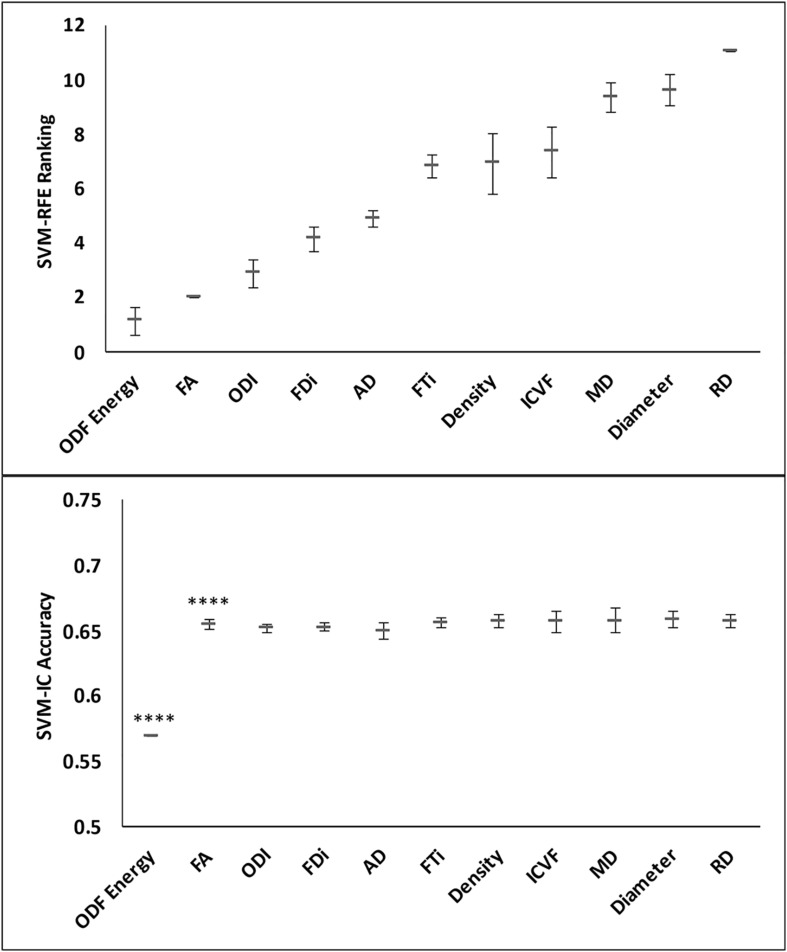
Feature ranks and classification accuracy in lesion-NAWM analysis. **Top panel:** The mean (standard deviation) rankings of the 11 diffusion features based on the linear recursive feature elimination (RFE) algorithm in SVM. **Bottom panel:** Accuracy of the classification models (SVM-IC) obtained by adding features one at a time, starting from the highest to the lowest rankings. The stars indicate features that contribute to significant improvement in classification accuracy (^****^p < 0.0001).

**FIGURE 6:**
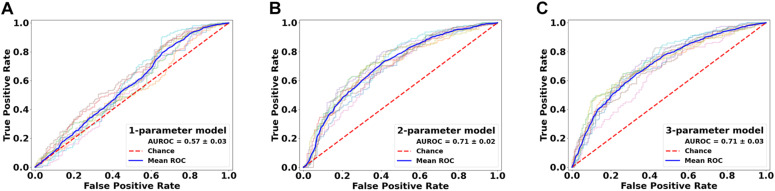
Performance comparison of the classification models in lesion versus NAWM analysis. Shown are the ROC curves for models constructed from the top three ranked diffusion features: **(A)** orientation distribution function (ODF) energy alone, **(B)** ODF energy + fractional anisotropy (FA), and **(C)** ODF energy + FA + orientation dispersion index. ROC, receiver operating characteristics; AUROC, area under the ROC curve.

Further assessments using the classification model revealed similar trends. Essentially, combining all top three features (ODF energy, FA, and ODI) in the SVM-IC model achieved a 0.65 accuracy and 0.71 AUROC (Figures 5 and 6). McNemar tests showed that model accuracy with ODF energy alone significantly outperformed chance (*p* < 2.2e-16) and improved further with addition of FA (*p* < 2.2e-16). ROC analysis mirrored these results. The AUROC was significantly better using ODF energy alone (*p* = 2.95e-6) than chance and improved with FA (*p* < 2.2e-16). Further, the AUROC peaked at 0.71 with both ODI and FDi added, but did not change significantly with further inclusion of the remaining features (Table 3). Model accuracy peaked at 0.66 with all but the lowest-ranked feature (DTI RD) included.

**TABLE 3:** The AUROC outcome of the linear SVM classification models.

**Features**	**Lesion vs. NAWM**		**Features**	**Core vs. Shell**	
	**AUROC**	**stdev**		**AUROC**	**stdev**
ODF energy	0.568	0.030	MD	0.594	0.080
FA	0.707	0.023	RD	0.597	0.088
ODI	0.710	0.026	FA	0.598	0.092
FDi	0.713	0.026	ODF energy	0.600	0.083
AD	0.710	0.029	AD	0.594	0.081
FTi	0.711	0.028	ICVF	0.595	0.081
Density	0.710	0.028	Density	0.598	0.082
ICVF	0.711	0.028	ODI	0.595	0.086
MD	0.710	0.028	Diameter	0.593	0.086
Diameter	0.710	0.028			
RD	0.710	0.028			

*AUROC, area under the receiver operating characteristic curve; stdev, standard deviation.*

### Core-Shell Analysis

Feature ranking in this assessment used 9 of the 11 diffusion features, with tractography FDi and FTi excluded due to their sparse representation in relatively small ROIs. The top three ranked metrics were: DTI MD, RD, and FA. HARDI ODF energy and DTI AD followed in ranking, slightly better than the other HARDI measures, which ranked at 6th–9th (ICVF, Density, ODI, and Diameter).

**FIGURE 7:**
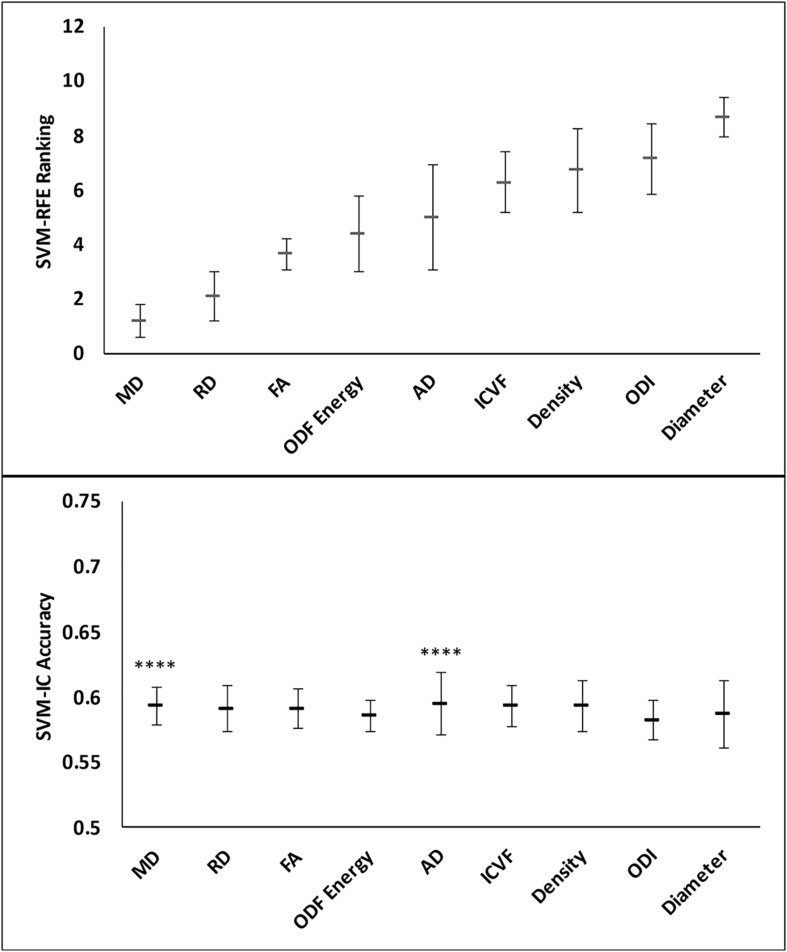
Feature ranks and classification accuracies in lesion core-shell analysis. **Top Panel:** The mean (standard deviation) rankings of the nine diffusion features used in this analysis based on the linear recursive feature elimination (RFE) algorithm in SVM. **Bottom Panel:** Accuracy of the classification models (SVM-IC) achieved by adding features one at a time, starting from the highest to the lowest rankings. The stars indicate features that contribute to significant improvement in classification accuracy (^****^p < 0.0001).

**FIGURE 8:**
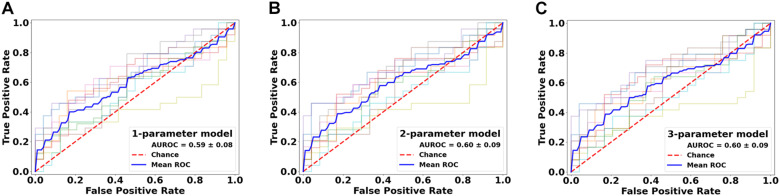
Performance comparison of the classification models in the lesion core versus shell analysis. Shown are the ROC curves for models constructed from the top three ranked diffusion features: **(A)** mean diffusivity (MD) alone, **(B)** MD + radial diffusivity (RD), and **(C)** MD + RD + fractional anisotropy. ROC, receiver operating characteristics; AUROC, area under the ROC curve.

Classification analysis further revealed the importance of top-ranked metrics in core-shell analysis. The highest-ranked feature, MD, alone accounted for a classification accuracy of 0.59, significantly greater than chance (*p* = 4.98e-5; Figures 7 and 8). Adding features up to the fifth one (AD), the classification accuracy peaked, at 0.60 (*p* = 3.86e-6). In addition, the model with MD alone achieved an AUROC of 0.59, which improved to the peak at 0.60 when combining with the top 2nd to 4th features (RD, FA, ODF energy), but the AUROC values were not significantly different from chance (*p* = 0.088; Table 3).

### Lesions and NAWM Analyses Between Subjects

**FIGURE 9:**
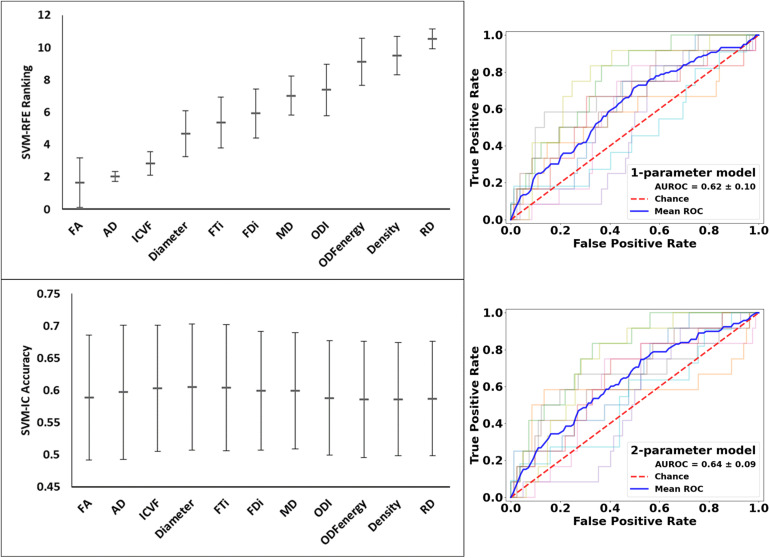
Feature ranks and performance comparison in the classification of lesion tissue between high and low disease patients. **Top Left Panel:** The mean (standard deviation) rankings of the 11 diffusion features used in this analysis based on the linear recursive feature elimination (RFE) algorithm in SVM. **Bottom Left Panel:** Accuracy of the classification models (SVM-IC) achieved by adding features one at a time, starting from the highest to the lowest rankings. **Right Panel:** ROC curves for models constructed using the top two ranked diffusion features: (top fractional anisotropy (FA) alone, and bottom) FA + axial diffusivity (AD). ROC, receiver operating characteristics; AUROC, area under the ROC curve.

**FIGURE 10:**
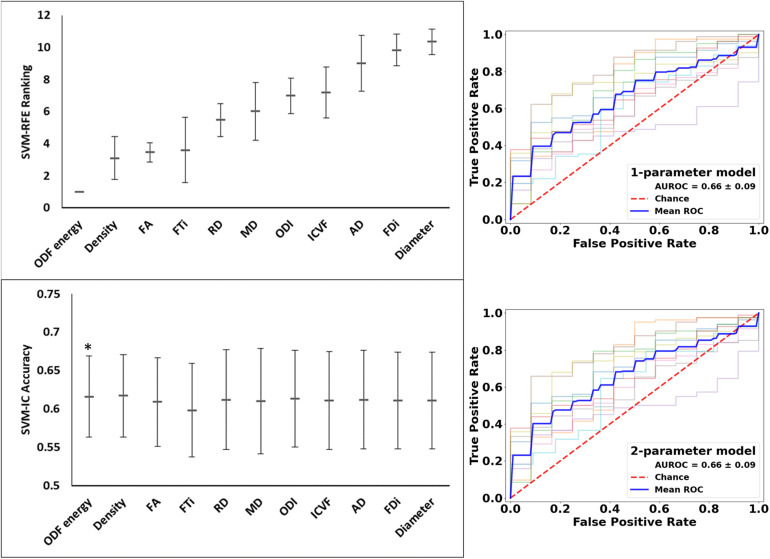
Feature ranks and performance comparison in the classification of NAWM tissue between high and low disease patients. **Top Left Panel:** The mean (standard deviation) rankings of the 11 diffusion features used in this analysis based on the linear recursive feature elimination (RFE) algorithm in SVM. **Bottom Left Panel:** Accuracy of the classification models (SVM-IC) achieved by adding features one at a time, starting from the highest to the lowest rankings. **Right Panel:** ROC curves for models constructed using the top two ranked diffusion features: [top orientation distribution function (ODF) energy alone, and bottom] ODF energy + Density (AD). ROC, receiver operating characteristics; AUROC, area under the ROC curve (^*^p < 0.05).

These analyses used all of the 11 diffusion features as applied in assessing whole lesion pathology. In comparing the lesion tissue between subjects who had 75%^*ile*^ high versus 25%^*ile*^ low lesion load, FA ranked the highest, which alone had an accuracy of 0.589 and AUROC of 0.620. Combining FA with the 2nd–4th features, AD, ICVF, and Diameter, slightly increased the accuracy, which reached the peak at 0.605; further addition of FTi as the fifth feature led to the peak AUROC of 0.638, but no models performed significantly better than chance (*p* = 0.200) in this analysis. In contrast, ODF energy ranked the best in classifying the matching NAWM regions between the same patient groups. Specifically, ODF energy alone achieved a near-peak accuracy of 0.616 and AUROC of 0.662, considerably better than chance (*p* = 0.020). Adding the second best parameter, neurite density, achieved the peak values for accuracy at 0.617 and for AUROC at 0.662, almost identical to the ODF energy alone results. There was no further improvement with addition of any other parameters (Figures 9 and 10).

## Discussion

Using commonly available dMRI data, we showed the feasibility of conducting ssHARDI analysis and the complementary value of parameters from relatively simple and complex diffusion models for assessing MS pathology. It appears that tissue alignment and orientation measures from ssHARDI and DTI are particularly sensitive to the existence of lesions in a patient, and to subtle structural differences between NAWM areas (specifically the ODF energy metric) between patients with high versus low disease activity. In contrast, tissue diffusivity and alignment metrics from DTI are the best in distinguishing intra-lesion pathology, and in separating lesion activity between high and low disease patients, especially MD and FA.

The ability to evaluate sub-voxel-based tissue properties through ssHARDI modeling would be important in a clinical setting. However, ssHARDI studies often face the challenge of not obtaining enough diversity of measures for evaluation, unlike multi-shell models. To compensate, a prior study applied a partial, 2-compartment model and showed that both neurite density and dispersion indices from ssHARDI decreased in the NAWM of MS patients, and the reduction in the right internal capsule correlated with MS duration (Magnollay et al., 2014). In the present study, we undertook an alternative approach with the assistance of an efficient modeling method, AMICO. This provided several sub-voxel measures of neurite diameter, density, and dispersion. Both visual and quantitative analyses show that the variances at a ROI level are equivalent between these measures derived using either 1-shell or multi-shell data, and using either online or own DTI data in ssHARDI modeling. In addition, based on the distribution of diffusion at all sampling directions in a voxel, we also created a new measure of tissue organization, ODF energy, to increase the capacity of ssHARDI.

To maximize the understanding of diffusion metrics in tissue assessment, our study considered several modeling approaches, 3 regarding ssHARDI alone: ActiveAx, NODDI, and diffusion ODF. Multi-model analysis is critical for assessing complex pathologies as observed in MS (Reich et al., 2018). However, this strategy also led to a large volume of parameters. A caveat of multiparametric approaches (Cercignani and Bouyagoub, 2018) is the difficulty of assigning importance to parameters that have overlapping sensitivities. Here we took advantage of SVM, a well-recognized machine learning method. The SVM-RFE has shown to be robust to feature redundancy and model overfitting, and in a linear form, the SVM can assign unique coefficients to each parameter, thereby identifying the specific contribution of a parameter to individual classification tasks (Guyon et al., 2002).

In lesion–NAWM analysis, nearly all top rankings were tissue orientation and alignment metrics, particularly the top 3: ODF energy, FA, and ODI. Based on definition, ODF energy measures the orientational complexity of a structure. The higher the value, the more misalignment in the structure. FA detects changes in both axonal density and alignment anisotropy (Zhang et al., 2012), and therefore is partially explainable by ODI. The leading performance of these orientation features in this analysis may indicate that the most critical changes following lesion formation in MS are tissue damage, such as inflammatory demyelination and axonal injury. A direct consequence of this pathology is increased structural heterogeneity, rather than simple alterations in neurite density. This is consistent with prior findings showing decreased FA in nearly all lesion studies in MS compared to the NAWM (Schmierer et al., 2007; Inglese and Bester, 2010), and ODI changes with lesion formation (Grussu et al., 2017; Schneider et al., 2017) and repair (Luo et al., 2019), although the consistency of ODI changes deserves further validation (Spano et al., 2018). Notably, optimal classification between lesion and NAWM ROIs in the current study required the addition of FA. Previously, tractography FDi also showed strong correlations with FA (Roberts et al., 2005; Ukmar et al., 2012), supporting the relationship between these highly ranked features. The unique role of tissue alignment and orientation measures may facilitate early detection and even prediction of lesion pathology in future studies, promoting early management.

The core-shell analysis was designed to probe the intra-lesion patterns of pathology as seen in chronic MS lesions. When active, these lesions show demyelinated, hypocellular cores and inflammatory demyelinating shells; while inactive, present with hypocellularity with no active demyelination in lesion territories (Pittock and Lucchinetti, 2007). As such, the expected differences between core and shell are the degree of cellularity, where the hypocellular core should have higher diffusivity than the cell-rich, inflammatory shell. Indeed, the 3 DTI measures (MD, RD, and FA) ranked the highest in our core-shell analysis, and MD played a dominant role. While there is lack of core-shell studies using dMRI in the literature, prior evidence attests the sensitivity of MD to subtle changes in MS pathology. One report showed that MD increased 5 months before the occurrence of active MS lesions, and pre-lesion MD correlated significantly with the MD of the lesions 2 months after active inflammation (Liu et al., 2012). In the present study, the lack of significance of the MD model in AUROC compared to chance may be due to several reasons, including the small number of such lesions and their heterogeneity in pathology. For instance, chronic inactive lesions may not show significant core-shell differences, and their inclusion may have caused artifacts. Alternatively, robust analysis of core-shell activity in chronic active lesions can improve our understanding of the ongoing pathology and so disease progression in MS patients (Giorgio et al., 2014).

Our inter-patient analysis results appear to agree with the findings described above. Between patients who have high versus low disease activity, FA ranked the highest in lesion tissue classifications; while in NAWM classifications, ODF energy was the best. In general, T2 MRI lesion load reflects disease activity, and in a long-term, it correlates with patient disability in MS as seen after 20 years of disease onset (Fisniku et al., 2008). Therefore, patients with higher lesion load are expected to have greater tissue damage, likely affecting both neurite density and orientation, and so greater changes in lesion FA between the patient groups. On the other hand, increased disease activity would also implicate increased pathology in the lesion-free areas, such as the NAWM, yet the changes wherein should be much less than in the visible plaques. Indeed, our NAWM analysis suggests that the pattern of differences between high and low disease patients is mainly orientation based, as reflected by ODF energy, deserving further confirmation.

Collective observations of this study may suggest that MD and FA are important diffusion metrics for assessing MS pathology. In particular, they may serve as top options in core versus shell analysis, disease severity comparisons between patients (e.g., lesion FA), and at a lesser extent, lesion versus non-lesion analysis. However, parameters from the relatively complex models appear to be more competitive in NAWM-associated evaluations than the DTI indices, particularly the orientation-driven metrics (ODF energy and ODI), suggesting the value of multi-model analysis. Currently, dMRI is one of the unique imaging approaches that can potentially evaluate both myelin and axonal properties *in vivo*. However, a variety of other techniques are underway in this regard, including those for myelin mapping using either advanced or conventional MRI. For example, myelin estimation based on simultaneous T1/T2 relaxometry and proton density mapping correlated strongly with histological myelin as seen in post-mortem MS brain samples (Ouellette et al., 2020). Another study demonstrated that quantitative susceptibility provided additional myelin information in brain NAWM of MS patients, independent of FA and RD (Yu et al., 2019). Further, pre-operative myelin mapping using T1/T2 ratio showed the potential to predict outcomes of trigeminal neuralgia following Gamma knife radiosurgery, while FA and RD demonstrated similar values in this prediction as estimators of pre-operative axons (Li et al., 2020). These findings further support the importance of multi-dimensional analysis in MS and similar diseases, including combining multi-model dMRI, and various other candidate metrics of myelin and axons.

We note a few limitations in this study. The spatial resolution of our dMRI was moderate. However, we registered all diffusion outcomes to the high-resolution T1 MRI to mitigate the impact, and our ssHARDI maps appeared similar to those obtained using high resolution dMRI from the HCP both visually and quantitatively. Next, to optimize data quality, we excluded lesions that did not have a “clean” match of contralateral NAWM regions, and lesions not large enough for core-shell analysis, limiting the sample size. Nonetheless, SVM analyses enabled determination of critical parameters, so the risk of model overfitting is minimal. Finally, there were lesions that appeared overlapping and they were segmented as single confluent regions. Given the inhomogeneity of signal intensity in such lesions, it may have somewhat affected the lesion-NAWM results (e.g., underestimation of tissue differences). However, we applied ROI means in all quantitative analyses, which should have minimized the effect, if any. The impact on core-shell analysis is expected to be less than the above, as regions of inhomogeneity may be included in both lesion portions. In the future, we seek to use higher resolution DTI and evaluate patients with different types of diseases and lesion pathologies to confirm our findings, including datasets with histology-verified lesion activity. Additionally, we also plan to investigate the utility of the top-performing diffusion features in assessing pre-lesion NAWM pathologies, and in predicting the occurrence of MS lesions, to promote clinical applications.

Collectively, using commonly available dMRI data, it is possible to perform competitive ssHARDI modeling. Combining machine learning with robust ssHARDI and DTI metrics may provide advanced assessment of lesion and NAWM pathology, including mean diffusivity for intra-lesion pathology in MS and similar diseases.

## Data Availability Statement

The original contributions presented in the study are included in the article/supplementary material, further inquiries can be directed to the corresponding author.

## Ethics Statement

The studies involving human participants were reviewed and approved by the Conjoint Health Research Ethics Board, the University of Calgary. The patients/participants provided their written informed consent to participate in this study.

## Author Contributions

OO participated in study design, data analysis and interpretation, and manuscript drafting and editing. W-QL participated in study design, data acquisition and analysis, and manuscript editing. BP, MK, and LM participated in study design, data acquisition, and manuscript editing. YZ participated in study design, data acquisition and interpretation, and manuscript drafting and editing. All authors contributed to the article and approved the submitted version.

## Conflict of Interest

The authors declare that the research was conducted in the absence of any commercial or financial relationships that could be construed as a potential conflict of interest.
